# Ultrasonic Transmission Tomography Sensor Design for Bubble Identification in Gas-Liquid Bubble Column Reactors

**DOI:** 10.3390/s18124256

**Published:** 2018-12-04

**Authors:** Nan Li, Mingchen Cao, Kun Xu, Jiabin Jia, Hangben Du

**Affiliations:** 1 School of Automation, Northwestern Polytechnical University, Xi’an 710129, China; xiaobendu@mail.nwpu.edu.cn; 2 Agile Tomography Group, School of Engineering, University of Edinburgh, Edinburgh EH9 3FG, UK; jiabin.jia@ed.ac.uk; 3 School of Mechanical Engineering, Xi’an Jiaotong University, Xi’an 710049, China; mingchen.cao@stu.xjtu.edu.cn; 4 Department of Applied Nuclear Technology, China Institute of Atomic Energy, Beijing 102413, China; 13716281629@163.com

**Keywords:** ultrasonic transmission tomography, sensor design, bubble distribution, bubble distribution, bubble column reactors

## Abstract

Scientists require methods to monitor the distribution of gas bubbles in gas-liquid bubble column reactors. One non-destructive method that can potential satisfy this requirement in industrial situations is ultrasonic transmission tomography (UTT). In this paper, an ultrasonic transmission tomography sensor is designed for measuring bubble distribution in a reactor. Factors that influence the transducer design include transmission energy loss, the resonance characteristics and vibration modes of the transducer, and diffusion angles of the transducers, which are discussed. For practical application, it was found that an excitation frequency of 300 kHz could identify the location and size of gas bubbles. The vibration mode and diffusion also directly affect the quality of the imaging. The geometric parameters of the transducer (a cylinder transducer with a 10 mm diameter and 6.7 mm thickness) are designed to achieve the performance requirements. A UTT system, based on these parameters, was built in order to verify the effectiveness of the designed ultrasonic transducer array. A Sector-diffusion-matrix based Linear Back Projection (SLBP) was used to reconstruct the gas/liquid two-phase flow from the obtained measurements. Two other image processing methods, based on SLBP algorithm named SLBP-HR (SLBP-Hybrid Reconstruction) and SLBP-ATF (SLBP-Adaptive Threshold Filtering), were introduced, and the imaging results are presented. The imaging results indicate that a gas bubble with a 3 mm radius can be identified from reconstructed images, and that three different flow patterns, namely, single gas bubble, double gas bubble with different diameters, and eccentric flow, can be identified from reconstructed images. This demonstrates that the designed UTT sensor can effectively measure bubble distribution in gas-liquid bubble column reactors.

## 1. Introduction

Gas-liquid bubble column reactors are widely used in the chemical industry. The gas phase is a discrete phase, while the liquid phase is a continuous phase, so bubble column reactors can be treated as two-phase flow reactors. Bubble column reactors are suitable for supporting slow chemical reactions or high heat release reactions. Bubbles are generated from the bottom of the column reactor, and these gas bubbles will aggregate and break apart during the ascent. The presence of bubbles accelerates the mixing between the chemical particles that are reacting, and this enhances the contact efficiency between the reaction objects, and improves the mass transfer and heat transfer effects. The size, distribution, and movement of the bubbles will affect the heat transfer and chemical reaction rates, which play an important role in the overall reaction performance. The behavior of the gas bubbles can be evaluated by measuring the gas concentration, the bubble velocity, and the size and distribution of bubbles of varied sizes across the reactor when viewed in cross-section [1,2]. A literature review indicated that many different methods have been applied to measure gas bubble distribution in bubble column reactors, and that this has been achieved with varying degrees of success. The methods used for measurement of gas bubble distribution can be classified into two categories: invasive techniques and non-invasive techniques. Typical invasive techniques include installing pairs of intrusive conductimetric electrodes [3,4,5] or needle type electrodes [6,7]. These methods operate within the reactor, which places limits on their suitability according to the measurement environment and the application conditions.

The pressure method and the tomographic technique are considered to be two typical non-invasive methods suitable for bubble behavior measurement. The pressure method has a simple working principle and is easy to implement [8,9]. The method is suitable for measuring average gas holdup in the sensing area, but it is difficult for it to detect the specific behaviors of bubbles in the local area, for example, details of bubble motion and the sectional distribution of the gas bubbles in the bubble column reactor. Tomography is a more effective non-invasive visualization technique. The distributions of objects in the sensing area can be reconstructed by using measurement data. The study of tomography began in the 1950s and two first-generation computerized tomography machines were developed in 1963 and in 1967 [10,11]. After decades of development, the process tomography (PT) technique became one of the most popular methods used in industrial process monitoring. For the analysis of gas holdup in bubble column reactors, X/gamma-ray computerized tomography [12,13], electrical resistance tomography (ERT) [14,15], and electrical capacitance tomography (ECT) [16,17,18] were also developed and are widely applied techniques. Some researchers also focused on using a combination methods to detect the behavior of gas bubbles in multiphase flow patterns. For examples, ERT technique coupled with differential pressure measurement to [19,20], and the ECT technique coupled with the optical measurement method [16]. Ultrasonic tomography (UT) can also be used to monitor the gas holdup in bubble columns [21,22] and other types of chemical mixtures, such as liquid-liquid flow patterns [23,24]. The working principle of ultrasonic tomography (UT) is based on the interaction between the ultrasonic wave and the medium measured. It has the advantages of creating no radiation risk, using relatively low cost sensors, and operating within a wide range of available frequencies. There are three sensing modes: transmission, reflection and diffraction mode [25]. Transmission mode and reflection mode are the most commonly used modes in UT systems, and they are usually referred to as ultrasonic transmission tomography (UTT) and ultrasonic reflection tomography (URT), respectively [26,27,28]. In 1996, Hoyle discussed the basic principles and advantages and disadvantages of using ultrasonic sensors for tomography, and presented the hotspots and difficulties that may be studied in the future of UT systems [29]. In next year, he and Li proposed a spectrum analysis method to extract the phase information of the received signal. The simulation proves that using a small number of sensors improves the acquisition speed of real-time data [30]. In 1999, Yang and Schlaberg used a relatively small number of sensors to excite the fan beam for real-time image reconstruction with a reconstruction speed of 100 frames per second. The effect of the reduction in the number of sensors on the imaging results was discussed [31].

This work aims to analyze the relation between influencing factors and design parameters of the ultrasonic transducer during sensor design, which is important for the final image reconstruction of the UTT system. The working principles and theoretical background are introduced firstly. Then, ultrasonic transducers are designed according to requirement of industrial application and recommend of influent factors of the ultrasonic transducer. The designed transducers are implemented and used to detect three typical bubbly flows. Finally, the performance evaluation of the designed transducers are provided.

## 2. Working Principles and Theoretical Background

**Figure 1:**
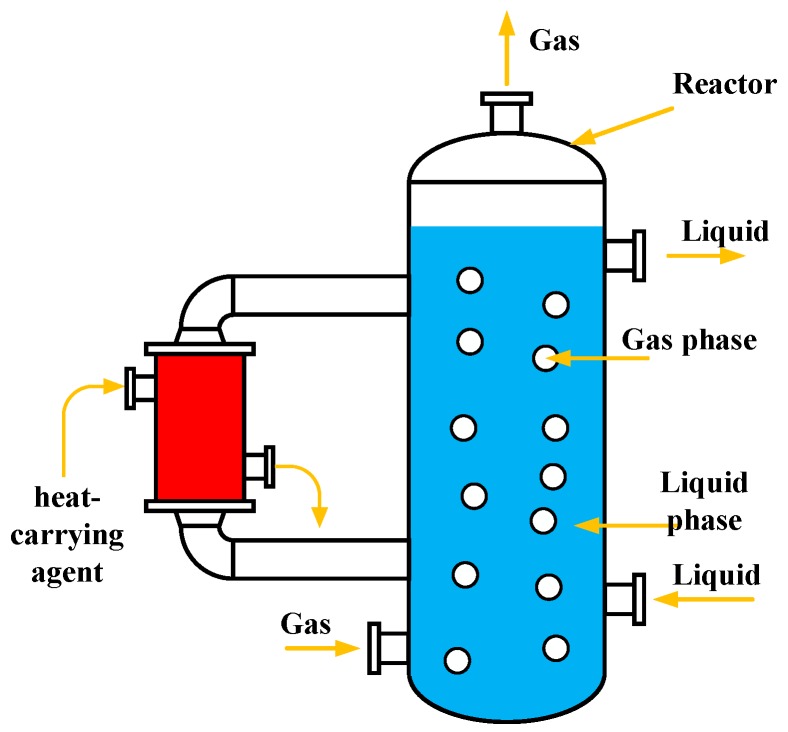
Typical structure of a bubble column reactor.

A typical bubble column reactor is shown in Figure 1. The liquid flows in from a pipe attached to the lower right of the reactor and flows out of the reactor from a pipe attached to the upper right part of the vessel. Gas flows in from the pipe attached to the lower left side of the reactor and flows out of the pipe attached at the top of the vessel. The separate component of reactor attached to the left side of the reactor is used to inject heat through a carrying agent, which can adjust the temperature of the reaction. A gas-liquid two-phase flow (bubbly flow) is then formed in the reactor. Once the reaction is underway, it is important to monitor the distribution of the bubbles in the reactor, which can have a significant influence on the reaction process and its effects.

**Figure 2:**
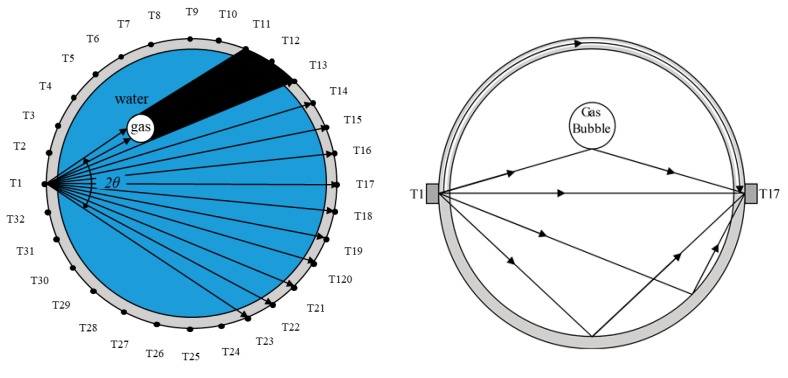
Working principle of ultrasonic transmission tomography and a schematic diagram of the path of sound propagation in a reactor.

A 32-transducer UT sensor is selected as an example. An array of 32 ultrasonic transducers (T1 to T32) is evenly placed around the bubble column reactor. The pitch-catch method (transmission mode) and pulse-echo method (reflection mode) are the modes typically used in the UT system for gas-liquid flow imaging, and in this paper, a pitch-catch method (transmission mode) is considered. The signals received at the transducers from a transmitted wave are analyzed to determine whether there are any objects on the path between the transmitter and the receiver. The TOF (time of flight) and the amplitude of the direct wave can be used for image reconstruction. The basic working principle of the measurement method is shown in Figure 2. 

T1 to T32 are dual function ultrasonic transducers, i.e., each of them can be set as a transmitter or a receiver. When T1 is set as a transmitter, for example, the other transducers are receivers. T1 emits the ultrasonic wave, and the opposing transducers in a fan-shaped angle range are set in the receiving state. The effective number of receivers depends on the angular width of the main lobe of the transducer, which is an important factor for final image reconstruction. This is determined by the diffusion angle of the designed transducer. The details of this are discussed in the sensor design section. As can be seen in Figure 2, when a gas bubble exists in the path between T1 and T11, and T1 and T12, receivers T11 and T12 will not receive the directly transmitted signals. Each transducer is then excited as a transmitter in turn, so that enough data will be obtained for imaging.

### 2.1. Influential Factors in Transducer Design for a UTT System

#### 2.1.1. Energy Loss during Propagation of Ultrasonic Waves

**Figure 3:**
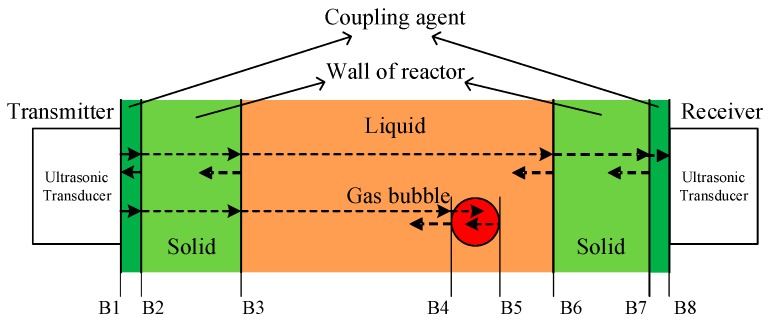
Transmission and reflection of ultrasonic wave at interface in gas liquid two-phase flow detection.

The actual propagation of ultrasonic waves between transmitter and receiver is, however, a complex process. For a typical bubble column reactor as shown in Figure 3, there are up to seven boundaries (B1 to B7) during the propagation of an ultrasonic wave. The ultrasonic wave is first excited by the transmitter. B1 is the first boundary, between the transmitter and the coupling agent, that connects the transmitter to the reactor wall. B2 is the boundary between the coupling agent and outer wall of the reactor, B3 is the boundary between the reactor inner wall and the liquid in the reaction area, B4 is the boundary between the liquid and the gas bubble, B5 between the gas bubble and the liquid on the opposing side of the bubble, B6 is between the liquid and the inner wall of the reactor on the opposite side of the reactor, B7 is between the outer wall of the reactor and coupling agent on that side, and finally, B8 is between the coupling agent and the receiver. In this process of propagation, the ultrasonic wave undergoes many phases of reflections and refractions. The propagated energy of the wave experiences attenuation and absorption, which has a direct impact on the measured results. In Figure 3, the colors of different areas refer to the different degrees of energy loss during propagation of the ultrasonic waves in those materials. For the coupling agent and the solid wall of the reactor, for example, most of the energy can penetrate with only limited energy loss. For the other interfaces, the energy loss depends on the difference of the acoustic impedance on either side of the interface.

Since imaging requires a large amount of measurement data, the issue of energy loss during wave propagation is very important. Energy loss in ultrasonic waves during propagation is mainly due to two processes: (a) The reflection and transmission of ultrasonic waves across the incident interfaces, and (b) the attenuation and absorption of ultrasonic waves in the propagation medium.

**Figure 4:**
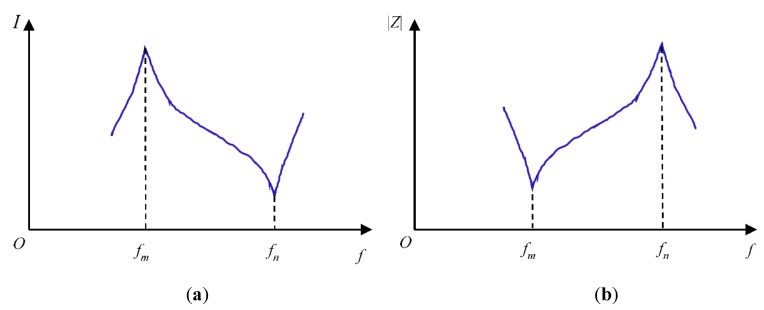
The relationship between internal current and input signal frequency of piezoelectric vibrator and its impedance curve. (**a**) The relationship between internal current and input signal frequency; (**b**) Impedance curve of piezoelectric vibrator.

The attenuation of sound waves discussed here is due to the characteristics of the propagation medium, including absorption and scattering attenuation, and it follows the law of exponential decay. As shown in Figure 4, attenuation of ultrasonic waves occurs during propagation through a medium, and this procession can be described by the following equation:

(1) $$p=p_{0}\exp(-\alpha L)$$

(2) $$\alpha =-\frac{1}{L}\ln\frac{p}{p_{0}}$$

where, *p* and *p*_0_ are the acoustic pressure after the propagation distance *L*, and the initial incident acoustic pressure, respectively, and *α* refers to the attenuation coefficient of sound pressure. At room temperature, attenuation coefficient in water α equals 25.3 × 10^−15^*f*^2^, and its unit is Np/m [22]. The attenuation coefficient α is proportional to the square of the excitation frequency *f*. The higher the frequency, the larger the attenuation coefficient α. Therefore, for an ultrasonic wave with a frequency of 300 kHz, after 100 mm of propagation in water, the pressure of the sound wave drops to 79.5% of its initial value.

#### 2.1.2. Resonance Characteristic and Vibration Modes of Transducers

The working frequency of the transducer is important, not only because the selection can affect the direction characteristic of the transducer, but it also directly influences the emitting power, efficiency, and sensitivity of the transducer. The equivalent impedance varies in proportion to the changes of frequency of the input signal. The minimum impedance frequency *f_m_* and the maximum impedance frequency *f_n_* correspond to the maximum conversion efficiency and the minimum conversion efficiency of the transducer respectively, which is shown in Figure 4. There are a series of resonance points for a transducer encountered when the frequency of the input signal keeps increasing. These critical points refer to other vibration modes including higher order vibration modes.

The vibration modes of the piezoelectric ceramic vibrator are determined by its polarization and excitation directions, and are also related to the geometry and size of the ceramic materials used. In this paper, a cylindrical piezoelectric vibrator is considered, and a longitudinal length extension vibration mode is required to produce a satisfactory vibration for the UTT system. In this case, Equation (3) can be used to describe the relationship between the antiresonance frequency *f*’ and the thickness of the piezoelectric ceramic vibrator *t*:

(3) $$f=\frac{n}{2t}\sqrt{\frac{c_{33}^{D}}{\rho }}=\frac{N_{t}}{t}$$

where *n* is natural number, which is 1, 3, 5, …, and $c_{33}^{D}$ is the elastic constant under constant electric displacement condition. *ρ* is the density of the piezoelectric ceramic. *N_t_* is the frequency constant in the thickness direction of the cylindrical piezoelectric vibrator. A radial extension vibration mode and a longitudinal length extension vibration mode may exist at the same time. To suppress any interference from a radial extension vibration mode on the longitudinal length extension vibration mode, the diameter of the vibrator should be much larger than its thickness.

#### 2.1.3. Diffusion Angle of the Ultrasonic Transducer

**Figure 5:**
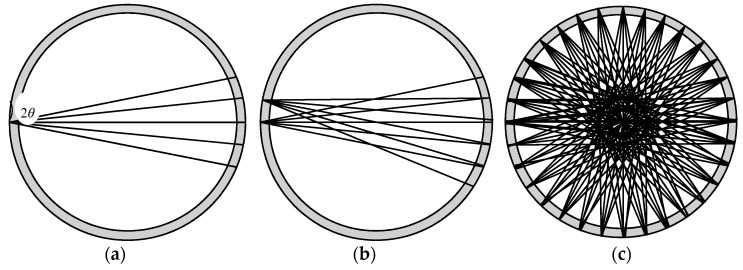
The schematic diagram of the superposition of acoustic waves emitted by sensors when the 2*θ* is 22.5 degrees (**a**) The sound beam width of a single sensor; (**b**) The superposition of the acoustic beam of two adjacent sensors; (**c**) The superposition of the sound beam of 32 sensors.

**Figure 6:**
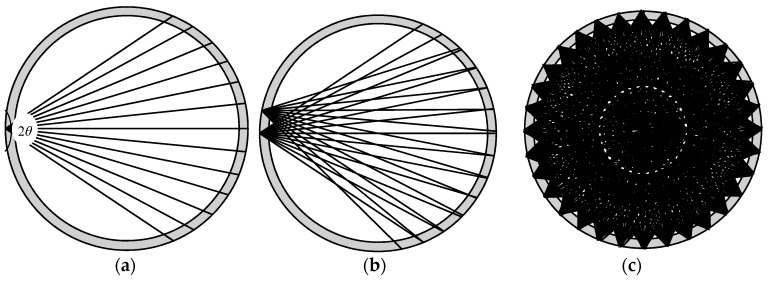
The schematic diagram of the superposition of acoustic waves emitted by sensors when the 2*θ* is 67.5 degrees (**a**) The sound beam width of a single sensor; (**b**) The superposition of the acoustic beam of two adjacent sensors; (**c**) The superposition of the sound beam of 32 sensors.

The ultrasonic wave emitted from a transducer propagates along a straight line within a certain angular sector range. The selection of the diffusion angle of the ultrasonic transducer depends on the specific detection purpose and its requirements. For image reconstruction with a 32-transducer UTT system, the wider the diffusion angle of a transducer, the more measurement values are available, which means better imaging quality. For example, a UT sensor may consist of 32 ultrasonic transducers with 22.5° diffusion angles. In this case, a signal is excited from a transmitter and the measurement signals are detected by four opposing receivers. Then, 32 transducers are set as exciters in turn, thus 128 measurements can be obtained. The ‘coverage’ of the sensing area is as shown in Figure 5. If 32 ultrasonic transducers are designed with 67.5° diffusion angles, and a signal is excited from a transmitter, then the measurement signals are detected by 12 opposing receivers. If 32 transducers of this configuration are set as exciters in turn, then 384 measurements can be obtained and the coverage of the sensing area is as shown in Figure 6. It is clear that the second UTT system arrangement has better ‘coverage’ of the sensing area than the first one. In other words, the wider the diffusion angle of the ultrasonic transducers, the more measurement data are obtained, which leads to better quality imaging results. The diffusion angle of the ultrasonic transducers is, therefore, an important factor for image reconstruction.

**Figure 7:**
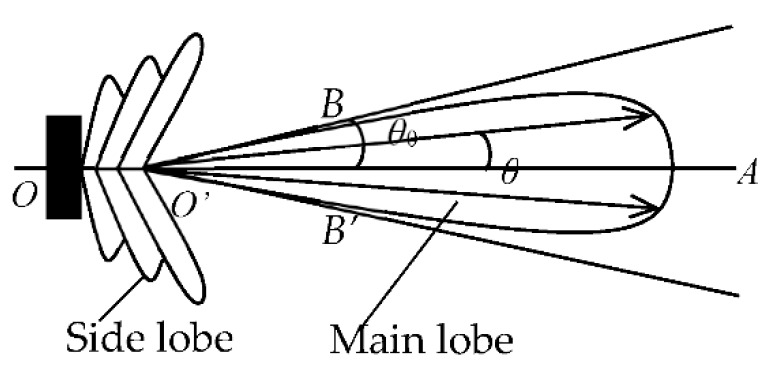
A schematic diagram of the diffusion angle of a piezoelectric wafer.

No matter whether it is set as transmitter or receiver, an ultrasonic transducer has a certain directionality. The directivity of the sound field is usually used to determine the energy distribution of the ultrasonic sound field. The majority of the energy is concentrated in a certain angular range centered on the acoustic axis. This range is the main sound beam, called the main lobe of the transducer. There are also low energy side beams named side lobes that emerge close to the source. The sound beam emitted by the transmitter gradually diffuses outward at a certain angle and its intensity is highest on the main axis. Figure 7 indicates a schematic diagram of the diffusion angle of waves from a piezoelectric wafer. The pressure *p* is a function of *l* and *θ*, where *l* is the distance from the measurement point to the excitation source, *θ* refers to the angle of the line connecting the measurement point and the excitation source, and OA is the central axis of the direction of propagation (Figure 7):

(4) $$p(l,\theta )=\frac{1}{l}\cdot \frac{J_{1}(ka\sin\theta )}{ka\sin\theta }=\frac{1}{l}\cdot g(\theta )$$

where *g*(*θ*) is a directivity function of the transducer, which is expressed as Equation (4), *J*_1_(x) is a first-order Bessel function. *k* is a constant corresponding to different degrees of attenuation (for example, −3 dB, −6 dB, or 12 dB), *a* is the radius of the ultrasonic wafer. If a circular piezoelectric wafer is taken as an example, half the diffusion angle *θ* can be calculated by Equation (6):

(5) $$g(\theta )=\frac{J_{1}(ka\sin\theta )}{ka\sin\theta }$$

(6) $$\theta =\sin^{-1}(\frac{k\lambda }{2a})$$

where, *λ* is a wave length of ultrasonic wave in the testing medium. The diffusion angle 2*θ*_−3dB_ denotes to the sum of the angles on both sides of transducer’s central axis (axis OA), and *θ*_−3dB_ is corresponding to the angel of the connection line between the point at −3 dB of the maximum response of signal (i.e., O’B and O’B’), and transducer’s central axis. 

Note that *k* is an empirical constant. *k* is 0.54 when −6 dB is considered, and *k* equals 1.08 when −20 dB is discussed. If a circular piezoelectrical transducer with a 10-mm diameter is taken as an example, the 2*θ*_−6dB_ and 2*θ*_−20dB_ diffusion angles of the transducer in water are 30.9° and 64.2°, respectively, when the excitation frequency is 300 kHz.

**Figure 8:**
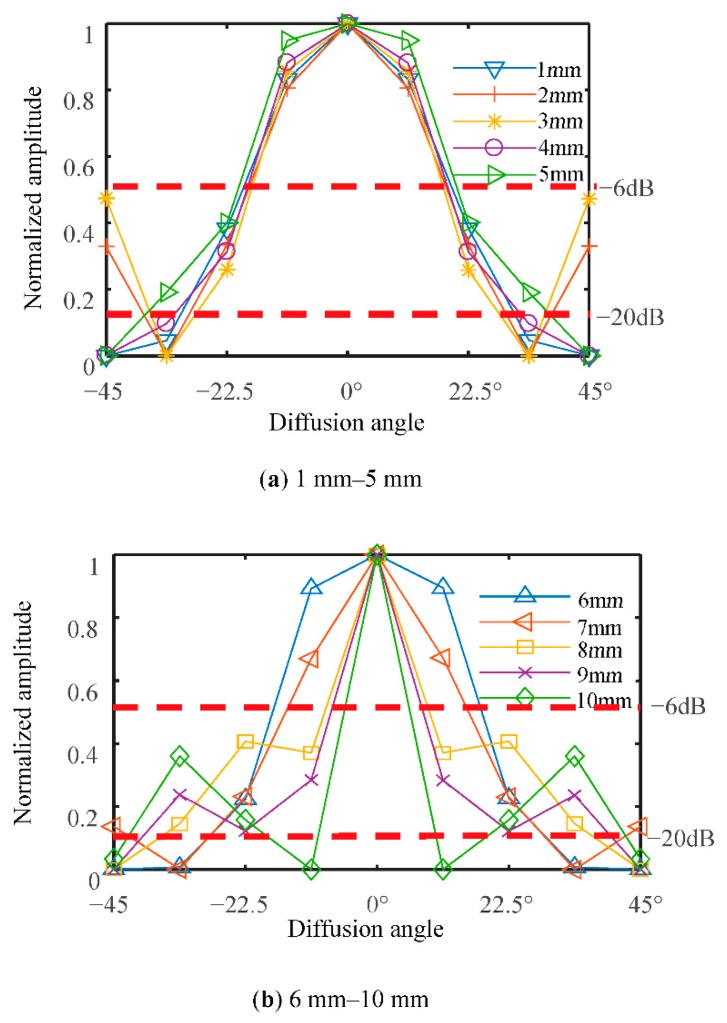
Directivity of different diameter piezoceramic cylinder.

A detailed graph simulating the relationship between the diameter of the transducer and its diffusion angle is presented in Figure 5. For a cylindrical reactor with a 110-mm diameter, the circumference is approximately 345 mm; therefore, the maximum diameter of an installed ultrasonic transducer is 10.7 mm for a 32-transducer UTT system. The frequency of excitation is set to 300 kHz, the reactor and the transducers are made of glass and PZT-5A ceramic material, respectively. The liquid phase in the sensing area is water. According to Equation (6), the half diffusion angle *θ* depends on the diameter of the transducer. The graphic shows plots for transducers with diameters at increments from 1 to 10 mm, at 1 mm intervals. In order to make the results clear to see, the 2*θ*_−6dB_ and 2*θ*_−20dB_ diffusion angles for the different diameter piezoceramic cylinders are shown in Figure 8a,b, respectively.

When the diameter of the transducer is greater than 5 mm, the width of the main lobe decreases as the diameter of the piezoelectric ceramic cylinder increases. When the diameter of the piezoelectric ceramic cylinder is less than 5 mm, the difference of main lobe width for different piezoelectric ceramic cylinder diameters is very small, i.e., as the diameter of the piezoelectric ceramic cylinder decreases, the main lobe width does not continue to increase. In order to have a wider diffusion angle, the diameter of the transducer should, therefore, be in the 5 to 10 mm range.

### 2.2. Linear Back Projection Algorithm for Image Reconstruction

The purpose of tomography can be described as reconstructing the profile and distribution of the detected object inside the detection area. There is a mapping relationship between the measured values at the sensors and the distribution in the object field, which can be expressed by the following equation. The inverse-solution of the problem can be calculated by Equation (8):

(7) $$\underset{n\times 1}{V}=\underset{n\times m}{S}\times \underset{m\times 1}{G}$$

(8) $$\underset{m\times 1}{G}=\underset{m\times n}{S^{-}}\times \underset{n\times 1}{V}$$

where ***V*** is the measurement vector, ***S*** is the coefficient matrix also called the sensitivity matrix, and ***S***^−^ is the inverse matrix of ***S***. ***G*** is referred to as the pixel vector of the medium distribution in the sensing area, *n* and *m* are the number of measurements and the number of pixels of the reconstructed image. Since the number of known quantities is fewer than the number of unknown quantities (*n* << *m*), and the inverse matrix of the coefficient matrix ***S*** does not exist (because ***S*** is not a full rank matrix), the inverse problem of the tomography is an ill-conditioned problem. In this case, ***S***^−^ should be replaced by a substitution matrix, or other iteration methods should be applied to calculate the matrix ***G***. For engineering applications, an algorithm named LBP (Linear back projection) is widely used, which employs a transposed matrix ***S****^T^* to replace ***S***^−^ in the calculation. Equation (9) then can be derived:

(9) $$\underset{m\times 1}{G}=\underset{m\times n}{S^{T}}\times \underset{n\times 1}{V}$$

**Figure 9:**
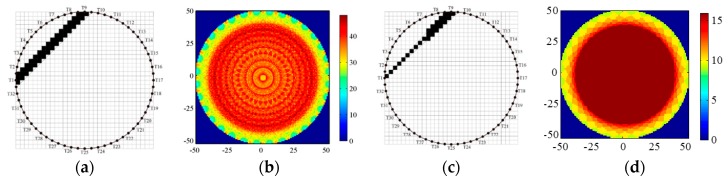
A schematic of a 32 × 32 rectangular array (**a**) a straight-line sensitivity matrix (**b**) The straight-line sensitivity matrices from all transducers (**c**) a sector sensitivity matrix. (**d**) The sector diffusion sensitivity matrices from all 16 transducers.

The cross-section of the imaging region is mapped onto 128 × 128 pixels consisting of two values 0 and 1 for water and gas bubbles, respectively. The sensitivity matrix ***S*** can be determined by summing all pixel values in the path of projections from one transmitter to receiver. 10mm width lines connecting transmitter to receiver form the area with pixel value 1. This area represents the straight-line sensitivity matrix as a schematic. Note that this is not applicable in the physical sense, but it is a common construction method for UT sensitivity matrix. For example, the cross-section of the imaging region is mapped onto 128 × 128 pixels, the straight-line sensitivity matrix from T1 to T9 is plotted in Figure 9a. An individual sensitivity matrix will be produced between any two transducers. As shown in Figure 9b, the final sensitivity matrices of all transducers are a superposition of these individual straight-line sensitivity matrices. The color-axis represents the pixel values. From Figure 9b, we can find there are many gaps in sensitivity matrix, that is to say, it cannot cover the whole imaging area, resulting in non-uniform pixel values.

Considering the covering ratio of the cross section, we next take the sector diffusion sensitivity matrix from T1 to T9 as an example (Figure 9c). This is produced by connecting those boundary points that are T1 and two midpoints between T8, T9, and T10. Compared to the final straight-line sensitivity matrix in Figure 9b, the full fan-shaped sensitivity matrix has better uniformity and continuity in Figure 9d, especially at the central area of the cross section.

## 3. Parameter Design and Implementation of the Transducers

During measurements, there are three principal transducer parameters that must be considered: the speed of sound in the medium, *c*, the wavelength, *λ*, and the frequency, *f*, of the transducer. Assuming an ideal gas bubble with a radius *r*, then in order to identify the bubble, *λ* should satisfy the condition *λ* << 2*πr*, considering that *λ* = *c/f* and *f* >> *c*/2*πr*. For gas-liquid two-phase flow detection, assuming the liquid phase is water and the gas phase is air, the minimum radius of gas bubble that can be detected by a 300-kHz ultrasonic wave is, in theory, 0.79 mm. According to the requirements of practical industrial applications, the minimum radius of gas bubble that must be detected is 3 mm, therefore, 300 kHz is adequate for these applications.

As discussed in Equation (3), the vibration mode of the transducer can be influenced by its geometry and size. In this work, the frequency constant in the thickness direction of the cylindrical piezoelectric vibrator, *N_t_*, of PZT-5A is 2000 Hz·m, and this is set by the manufacturer. The thickness, *t*, of the vibrator should, therefore, be 6.7 mm when the resonance frequency of the transducer is designed to be 300 kHz. In order to have a longitudinal length extension vibration mode, the diameter of the vibrator should be much larger than the thickness.

**Figure 10:**
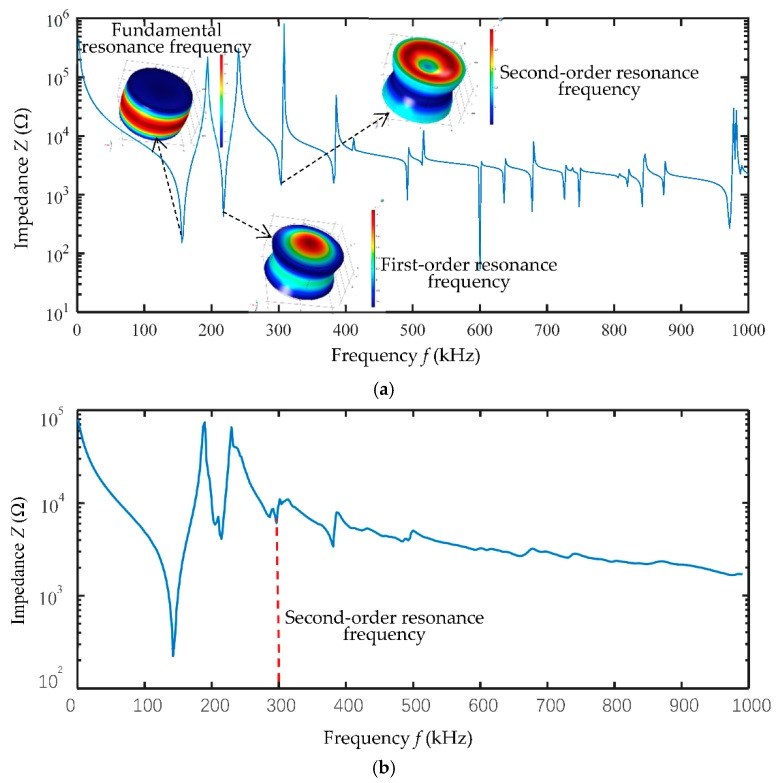
The impedance curve and the surface stress of the resonance frequency point of the piezoelectric cylinder with 10 mm diameter and 6.7 mm thickness.

To generate a longitudinal vibration mode at a resonance frequency of 300 kHz, the diameter of the transducer is set to 10 mm and the thickness of the transducer is set to 6.7 mm. The impedance curve and the surface stress at the resonance frequency point of the piezoelectric cylinder are shown in Figure 10a, and the fundamental, the first-order and second-order resonance frequencies are presented in the figure. A longitudinal vibration mode are generated at the second-order resonance frequency point which is 300 kHz. A batch of transducers were then produced and the impedance curve evaluated to verify the result, as shown in Figure 10b.

**Figure 11:**
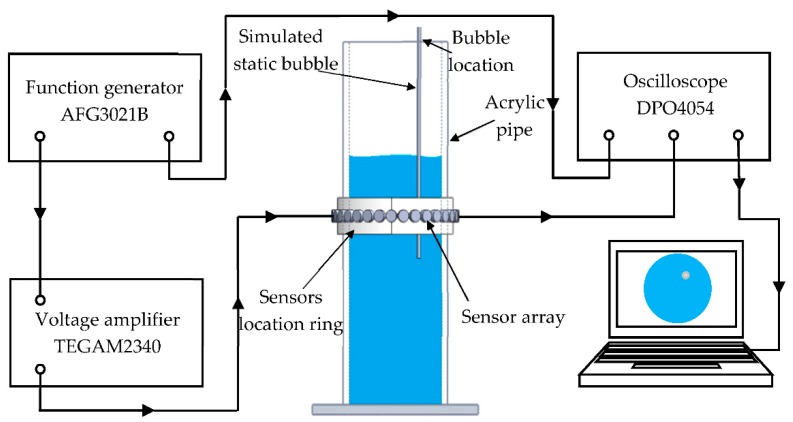
A schematic diagram of gas-liquid two-phase flow UT detection system.

An UTT system was established to test the performance of the designed transducer. As shown in Figure 11, the system consisted of a function generator (AFG3021B), an ultrasonic transducer array, a multiplexer, a voltage amplifier (TEGAM2340), a digital oscilloscope (DPO4054), and a PC. Due to limitation on the number of channels available in the multiplexer, 16 transducers were the maximum that could be applied to this UTT system. The experimental setup in the lab included a reactor made of glass with outer and inner diameters of 110 mm and 100 mm, respectively. The height of the reactor was 500 mm. The liquid phase was water and the gas phase were air This meant that the gas bubbles rose very quickly, so during the transducer performance testing stage, we used different sizes of empty pipes made of soft plastic and full of air as a proxy for gas bubbles in that location. In order to reduce the impact of the coupling agent, the ultrasonic transducers were fixed to the tube outer wall with 502 glue.

## 4. Experimental Results and Analysis

### 4.1. Experimental Method

**Figure 12:**
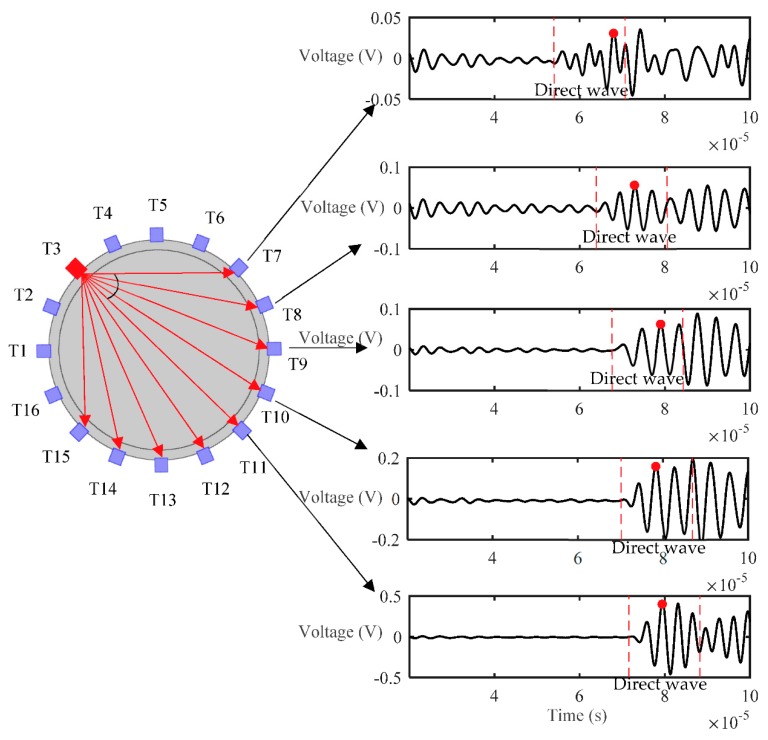
Time domain signal received by T7~T15 under full pipe.

**Figure 13:**
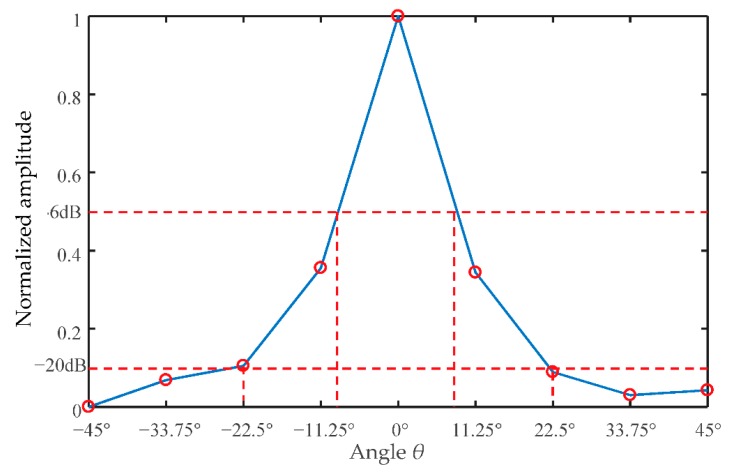
Transducer directivity by experimental measurement.

For the experiments, the excitation signal was a 5-cycle Hanning window modulated sine wave with a frequency of 300 kHz. A 3-volt excitation voltage was produced by the function generator, and then amplified by 50 times so that the voltage of the excitation at the transducer was 150 V. The signal sampling rate was 10 MHz, the number of sampling point was 1000, and the sampling duration was 100 μs. The signals obtained under full reactor conditions were determined to be the reference signal set. All of the transducers could be set as transmitters or as receivers, and each transducer was excited in turn. Figure 12 presents the arrangement of the transducers (T1 to T16) and received signals (within the 0–100 μs time period) at the opposing transducers (T7 to T15, which were within the diffusion angle range), when T3 was set as the transmitter. Note that only signals received by T7 to T11 are presented in Figure 12 because of the symmetrical structure. As shown in Figure 12, the amplitude of the direct waves received from T7 to T11 increased in steps because of the effects of the diffusion angle variation. The TOF (time of flight) of the direct wave from T3 to T7 through T11 gradually increased. As discussed in Section 2.1.3, the diffusion angles 2*θ*_−6dB_ and 2*θ*_−20dB_ of the transducer in water should, in theory, be 30.9° and 64.2°, respectively, however, the actual diffusion angles 2*θ*_−6dB_ and 2*θ*_−20dB_ of the designed transducers were 17.8° and 45°, respectively. The reason for this difference is that the diffusion angle of the transducer is affected by the thickness of the reactor’s wall. Note that only the signals received by T9 through T13 could be used to reconstruct images with the diffusion angle 2*θ*_−20dB_ of 45°. The normalized amplitude of the transducers is shown in Figure 13, which also indicates how many effective values were received by the transducers that could be used to reconstruct images.

Before the different flow-patterns were tested, the resolution of the designed transducer was verified. As mentioned in Section 3 a bubble with a 3-mm radius should, in theory, be easily identified by the designed transducers. Single simulated bubbles with different radiuses were set up as test cases to evaluate the resolution of the transducers. Table 1 presents two single bubbles with different radiuses of 3 mm and 5 mm, respectively. The imaging results indicated that the gas bubble with a 3-mm radius could be identified by the UTT system with the designed transducers. The bubble size can be qualitatively resolved.

**Figure 14:**
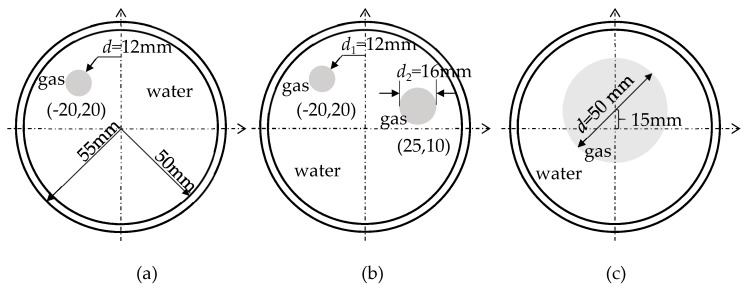
Three different experimental flow patterns. (**a**) single gas bubble (**b**) double gas bubbles with different diameters (**c**) eccentric flow.

Three different flow patterns, namely, single gas bubble, double gas bubbles with different diameters, and eccentric flow, were considered in following experiments. The geometric parameters of the bubbles and the reactor are shown in Figure 14. 

There were 8 and 16-transducer UTT systems that were used to detect and reconstruct these phantoms, respectively. Three different methods based on the LBP algorithm were used to evaluate the quality of the imaging results:

*Method 1*: The SLBP (Sector-diffusion-matrix based Linear Back Projection) is based on a traditional LBP algorithm, which uses a sector-diffusion-matrix to replace the traditional sensitivity matrix applied in UT image reconstruction [32].

*Method 2*: SLBP-HR (SLBP-Hybrid Reconstruction) is an imaging processing algorithm that uses an HR masking matrix to multiply the pixels of the original LBP image. A value equal to 3/4 of the maximum pixel value of the original LBP image was set as the threshold [33].

*Method 3*: SLBP-ATF (SLBP-Adaptive Threshold Filtering) is an image binarization algorithm that uses an adaptive filtering method to determine the best threshold value [34]. The imaging results based on these three algorithms are shown in Table 2.

### 4.2. Results and Discussion

Some conclusions can be drawn directly from the images in Table 2. (i) The designed transducers are good enough to reconstruct images, however, the image quality is affected by the number of transducers used. The more transducers, the better the image quality. (ii) For 45° diffusion angle transducers, the flow patterns can be recognized correctly when the number of transducers is 16. The sizes and the locations of the bubbles can also be identified for double gas bubbles. (iii) For 16-transducer UTT systems, the imaging results from double gas bubble models reconstructed using the SLBP-HR method are better than the imaging results constructed using the SLBP-ATF algorithm. For SLBP-HR method, the size and profile of double bubbles are closer to the real situation.

The image correlation coefficient, *I_cr_*, is defined to evaluate the quality of reconstruction images:

(10) $$I_{cr}=\frac{\sum_{i=1}^{m}\left({\hat{g}}_{i}-\bar{\hat{g}})(g_{i}-\bar{g}\right)}{\sqrt{\sum_{i=1}^{m}{\left({\hat{g}}_{i}-\bar{\hat{g}}\right)}^{2}\sum_{i=1}^{m}{\left(g_{i}-\bar{g}\right)}^{2}}}$$

where, ${\hat{g}}_{i}$ and $g_{i}$ refer to the ith pixel values of the reconstructed binary image and the standard images respectively. $\bar{\hat{g}}$ and $\bar{g}$ are the mean values of matrices $\hat{g}$ and *g*, respectively. Term *m* is the number of pixels. The calculation results are listed in Table 3.

## 5. Conclusions

In this research project, ultrasonic transducers were designed and tested within a UTT system in order to evaluate bubble distribution measurement methods for gas-liquid bubble column reactors. Some conclusions can be drawn from the results of the experiments as follows:

(i) The energy loss, the resonance characteristics and vibration modes, and the diffusion angle of the transducers were the three most critical factors for sensor design. Energy loss should be as low as possible in order to derive better response signal strengths. The resonance characteristics and vibration modes must be appropriate for the measurement setup and to achieve the measurement objectives. The diffusion angle should be as wide as possible so that more measured values can be obtained, for better imaging quality.
(ii) The diameter and thickness of the transducer directly affect the above three factors. For a reactor with a 100-mm diameter and a signal amplitude of 300 kHz, the ultrasonic wave strength will drop to 79.5% of the original value after 100 mm of propagation. To excite a longitudinal length extension vibration mode at 300 kHz resonance frequency, the diameter and thickness of the transducer should be 10 mm and 6.7 mm, respectively. The 2*θ*_−6dB_ and 2*θ*_−20dB_ diffusion angles of the designed transducers are 17.8° and 45°, respectively, and this is slightly different to the theoretical results for the 2*θ*_−6dB_ and 2*θ*_−20dB_ diffusion angles for the transducer in water, which should, in theory, be 30.9° and 64.2°. This discrepancy results in less measurement data collected, which will in turn affect the quality of the reconstruction images.
(iii) Three different flow patterns were considered. An SLBP algorithm was used to reconstruct images from the designed UTT system. The SLBP-HR and SLBP-ATF methods were applied to process the reconstructed images. An image correlation coefficient, *I_cr_*, was used to evaluate the quality of imaging results. The results indicate that, in addition to each sensor’s own performance, the total number of sensors also has an important impact on the imaging results. The greater the number of transducers used, the better the imaging quality.

The transducer designed in this project can be effectively used in a 16-transducer UTT system for bubble distribution measurement in gas-liquid bubble column reactors.

## Figures and Tables

**Table 1:** Reconstruction results of single bubble with 3 mm and 5mm radius by LBP algorithm.

*r*/mm	Phantoms	*r* = 2 mm	*r* = 5 mm
3	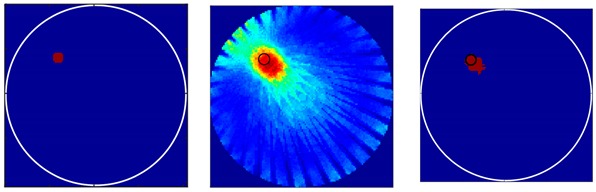		
5	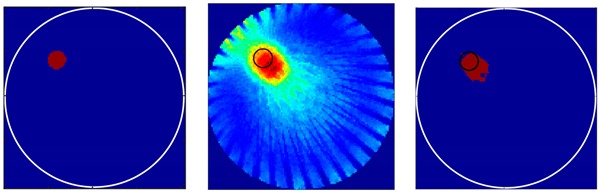		

**Table 2:** Reconstruction results of different flow patterns by three LBP-based methods.

Image Reconstruction Methods		Phantom	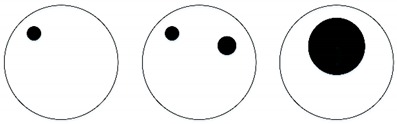	
	Number of Transducers			
**SLBP**	**8**		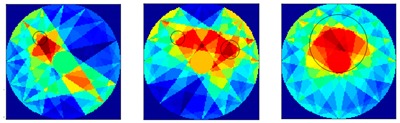	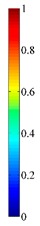
	**16**		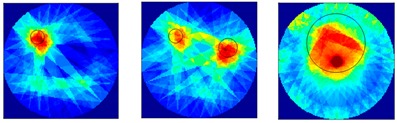	
**SLBP-HR**	**8**		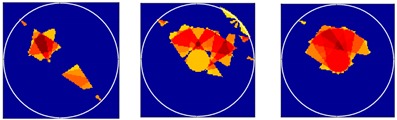	
	**16**		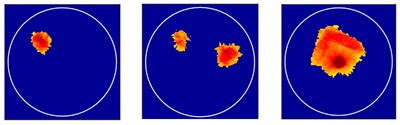	
**SLBP-ATF**	**8**		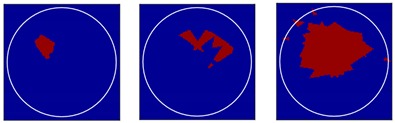	
	**16**		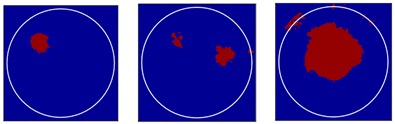	

**Table 3:** Evaluation indexes of reconstructed image after processing by FLBP-ATF method.

Image Correlation Coefficient	Reconstruction Algorithm	The Number of Transducers	Single Gas Bubble	Double Gas Bubbles	Eccentric Gas-Liquid Flow
*I_cr_*	SLBP-ATF	8	0.320	0.315	0.754
		16	0.698	0.579	0.708
